# Allocation Costs Associated with Induced Defense in *Phaeocystis globosa* (Prymnesiophyceae): the Effects of Nutrient Availability

**DOI:** 10.1038/srep10850

**Published:** 2015-06-04

**Authors:** Xiaodong Wang, Yan Wang, Linjian Ou, Xuejia He, Da Chen

**Affiliations:** 1 Research Center for Harmful Algae and Marine Biology, Jinan University, Guangzhou, 510632, China; 2 Key Laboratory of Eutrophication and Red Tide Prevention of Guangdong Higher Education Institutes, Jinan University, Guangzhou, 510632, China; 3 Cooperative Wildlife Research Laboratory and Department of Zoology, Southern Illinois University Carbondale, Carbondale, Illinois 62901, USA

## Abstract

Colony enlargement in *Phaeocystis globosa* has been considered as an induced defense strategy that reduces its susceptibility to grazers, but allocation costs inflicted by this plastic morphological defense are poorly understood. We conducted experiments in which *P. globosa* cultures were exposed to chemical cues from copepods, ciliates and heterotrophic dinoflagellates, respectively, under nutrient sufficient and deficient conditions to evaluate allocation costs associated with induced defense. *Phaeocystis globosa* responded to chemical cues from grazers by increasing colony diameter irrespective of nutrient conditions. We did not find trade-offs between induced defense and growth rate under nutrient sufficient conditions. Instead, induced defensive *P. globosa* had higher growth rates than non-induced *P. globosa*. When nutrient became limited, *P. globosa* exposed to grazing cues from copepods and dinoflagellates had significantly decreased growth rates when compared with non-induced *P. globosa*. We suggested that the decreased growth revealed allocation costs associated with induced defense that may influence on the trophic interactions between *Phaeocystis* and consumers.

*Phaeocystis*, a potential harmful algal species with worldwide distribution, plays important roles in trophic structure, biogeochemical cycles and climate change^1^,^2^. It has a unique polymorphic life cycle that involves transformation between solitary cells and gelatinous colonies. The solitary cells are generally 3-10 μm in diameter. The colonies, with cells distributed in a mucilaginous matrix, can reach up to 3 cm in diameter^3^,^4^. Colony formation is one of the remarkable plastic phenotype characters in *Phaeocystis*. Colony diameter increases significantly in the presence of grazers or chemical signals originating from grazing activities^5^,^6^,^7^. Because large colonies create a size-mismatch problem for small grazers^8^,^9^, such plastic morphological responses significantly reduce grazing mortality^5^. Colony formation and enlargement have been considered as induced defense strategies that reduce predation risk, and play key roles responsible for the success of *Phaeocystis* in marine systems^6^,^9^.

It is widely assumed that any physiological advantage must have a trade-off in metabolic costs^10^,^11^. Because defense and growth are energy demanding, limiting resources could be allocated to growth or reproduction under predation-free conditions to optimize the energy expenditures. In contrast, a prey organism in the presence of predator has to invest resources for defense, and consequently decrease growth^12^,^13^. The reduction in growth rate can be viewed as allocation cost associated with induced defense^14^,^15^. Studies on terrestrial plants have provided support for the existence of allocation cost. For example, the suppressed growth and photosynthesis, reduced seed production and leaf growth, and delayed flowering and fruiting have been associated to induced resistances to herbivores^16^,^17^,^18^.

Not all studies attempting to explore the costs of induced defense have been successful, particularly in the case of *Phaeocystis*. Although its induced defense against grazing has been well described^5^,^6^,^7^,^19^,^20^, understanding of the allocation cost is currently lacking. Tang^6^ showed that *P. globosa* responded to chemical cues from dinoflagellate *Gyrodinium dominans* by increasing both abundances of solitary and colonial cells, indicating that induced defense had positive influence on the growth. The presence of grazing chemical cues from natural zooplankton not only stimulated the colony enlargement, but increased the abundance of total cells of *P. antarctica*^7^. Similar studies by Long *et al*.^19^ also reported that chemical cues released by *Acartia* strongly enhanced the growth rate of *P. globosa*. One reason for failure to estimate allocation costs of inducible defense is that costs are strongly dependent on resource availability^14^,^15^. The allocation costs may be undetectable under conditions of high resource availability that allow for both active growth and effective defense. Costs become more apparent under resource-limiting conditions^14^,^21^,^22^. For example, *Chlorella vulgaris* under rotifer grazing pressure became smaller in diameter as a defense response, resulting in 32% lower rotifer growth rate relative to the ones feeding on non-defensive algae^23^. The induced defensive *C. vulgaris* exhibited lower growth rate than non-induced algae only when cultured in nitrate-deficient medium, but not in nitrate replete medium^23^. Accordingly, we predict that allocation costs of induced defense in *Phaeocystis* under low nutrient condition would increase and become detectable.

The goal of this study was to measure the expression and allocation costs of induced defense in *P. globosa*. We exposed *P. globosa* culture to grazing cues associated with copepods, ciliates and heterotrophic dinoflagellates under nutrient sufficient vs. nutrient deficient conditions. The growth rates were used as proxies of fitness, and colony diameters and percentages of cells in colonial form were measured as proxies for defensive response. Given the ecological roles of *Phaeocystis* in marine carbon and sulfur cycles, measurement of allocation costs associated with induced defense is important for understanding the structure and function of the marine systems dominated by *Phaeocystis*.

## Results

### Nutrient concentrations

Nitrate and phosphate concentrations in all grazing treatments and controls decreased consistently at the end of experiments (Table 1). Nitrate concentrations under HN and LN conditions were higher than 50 μM and 1.0 μM, respectively. Phosphate concentrations remained >0.5 μM under HN condition, but fell below the analytical detection limit under LN condition. The concentrations of NH_4_^+^ were <0.55 μM under both HN and LN conditions for all experiments.

### Grazing inside cages

Abundances of solitary cells in *P. globosa* inside all grazing cages were significantly lower than those inside control cages under both nutrient conditions as a result of predation by three grazers (Copepod, HN: *P = *0.0014, LN: *P = *0.0174; Ciliate, HN: *P = *0.0017, LN: *P = *0.0097; Dinoflagellate, HN: *P = *0.0005, LN: *P = *0.0001; Table 2). The final concentration of *Euplotes* sp. increased to 54.0 ± 8.2 cells mL^−1^ under HN condition and to 20.3 ± 6.5 cells mL^−1^ under LN condition. Similarly, *O. marina* concentration under HN and LN conditions increased to 1083.3 ± 60.5 cells mL^−1^ and 511.0 ± 101.7 cells mL^−1^, respectively. In contrast, abundances of copepod remained unchanged at the end of experiments.

### Induced defensive responses

**Figure 1:**
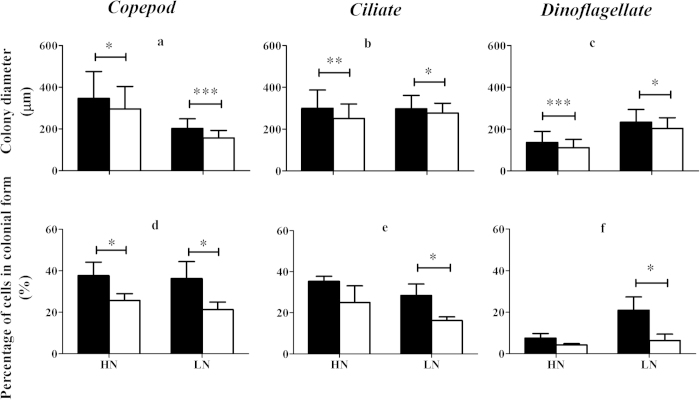
Colony diameters (top panel) and percentages of cells in colonial form (bottom panel) in *P*.*globosa* outside grazing (filled bars) and control (open bars) cages. HN and LN are high and low nutrient levels, respectively. Values are mean ± 1SD. ^*^ *P *< 0.05, ^**^ *P *< 0.01, ^***^ *P *< 0.001.

The presence of grazing cues strongly influenced colony size and partitioning of cells between colonial and solitary forms of *P. globosa* (Fig. 1). Colony diameters of *P. globosa* outside all grazing cages were more than 10% larger than those outside control cages, irrespective of nutrient conditions (Copepod, HN: *P = *0.0306, LN: *P = *0.0001; Ciliate, HN: *P = *0.0012, LN: *P = *0.045; Dinoflagellate, HN: *P = *0.001, LN: *P = *0.0399, Fig. 1 a-c). Exposure to grazing cues from copepods significantly raised the percentages of cells in colony form under both nutrient conditions (HN: *P = *0.0425, LN: *P = *0.0397, Fig. 1 d). Grazing cues from ciliates and dinoflagellates did not significantly change the partitioning of cells between colonial and solitary forms under HN condition (*P *> 0.05), but resulted in higher percentages of cells in colony form under LN condition (Ciliate: *P = *0.0195; Dinoflagellate: *P = *0.0454, Fig. 1 e-f).

### Proxies of fitness

**Figure 2:**
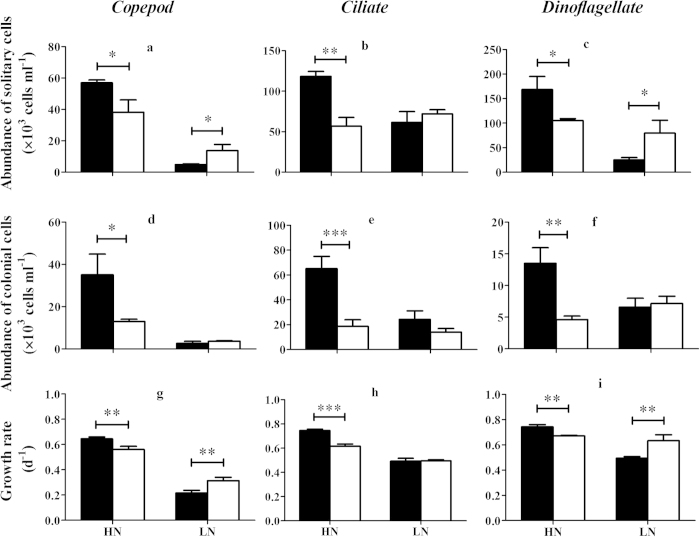
Abundances of solitary cells (top panel), abundances of cells in colonial form (middle panel) and population growth rates (bottom panel) in *P*.*globosa* outside grazing (filled bars) and control (open bars) cages. HN and LN are high and low nutrient levels, respectively. Values are mean ± 1SD. ^*^ *P *< 0.05, ^**^ *P *< 0.01, ^***^ *P *< 0.001.

Induced defensive *P. globosa* had higher abundances of solitary cells than non-induced *P. globosa* in response to chemical cues from all grazers under HN conditions (Copepod: *P = *0.0162; Ciliate: *P = *0.0010; Dinoflagellate: *P = *0.0147, Fig. 2 a-c). When grown under LN condition, however, the abundance of solitary cells of induced *P. globosa* was decreased in the presence of grazing cues from copepods and dinoflagellates relative to non-induced *P. globosa* (Copepod: *P = *0.0166; Dinoflagellate: *P = *0.0243). Exposure to ciliate grazing cues did not influence the abundance of solitary cell of induced *P. globosa* (*P *> 0.05).

Abundances of colonial cells of induced defensive *P. globosa* were up to 2-fold higher than those of non-induced *P. globosa* under HN condition (Copepod: *P = *0.0181; Ciliate: *P = *0.0006; Dinoflagellate: *P = *0.0038, Fig. 2 d-f). However, there was no statistically significant differences between induced and non-induced *P. globosa* under LN condition (*P *> 0.05).

The growth rates of *P. globosa* were influenced strongly by nutrient conditions and grazing cues (Fig. 2 g-i). Grazing cues from all grazers resulted in increased growth rates of induced *P. globosa* when compared to the controls under HN condition (Copepod: *P = *0.0077; Ciliate: *P = *0.0004; Dinoflagellate: *P = *0.0029). However, growth rates of *P. globosa* exposed to grazing cues from copepods and dinoflagellates were significantly lower than those of non-induced *P. globosa* under LN condition (Copepod: *P = *0.0079; Dinoflagellate: *P = *0.0083). There were no differences in growth rate between induced- and non-induced *P. globosa* in ciliate experiments under LN condition (*P *> 0.05).

## Discussion

The major goal of this study was to measure the allocation costs of induced defense in *Phaeocystis globosa*, an important HAB species with a cosmopolitan distribution. With various defensive mechanisms, *P. globosa* has been considered as a model candidate for studies on the defense of primary producer in marine ecosystems^9^. Transparent exopolymer particles (TEP) and cohesion of the threads into pentagonal stars significantly reduced the vulnerability of *P. globosa* solitary cells to grazing copepods^24^,^25^. These defensive effects have important implications for increasing survival of solitary cells on termination of the colonial bloom^24^,^25^. There was no evidence indicating that the defense mechanism of solitary cells is inducible^24^. In contrast, the theory that *Phaeocystis* colony formation and size increase are induced defense mechanisms against grazers has been widely acknowledged^6^,^7^,^19^,^20^; see also^9^. Therefore, we have focused on the induced defensive responses of *P. globosa* colonies in the present work.

The assumed most important defense trait in *Phaeocystis* is colony enlargement that provides protection for colonial cells against small grazers due to size-mismatch and the tough colonial envelope^8^,^26^. Tang^6^ found that dissolved chemicals released during heterotrophic dinoflagellate or copepod grazing activities resulted in significant colony enlargement in *P. globosa*. The presence of grazing chemicals associated with zooplankton has also been shown to induce *P. antarctica* to form significantly larger colonies^7^. In this study, *P. globosa* exposed to grazing cues from copepods, ciliates and heterotrophic dinoflagellates developed consistently larger colonies than non-induced *P. globosa* irrespective of nutrient levels. The magnitudes of colony enlargement in LN treatment, which had a negative influence on growth rates of *P. globosa*, were similar to those in HN treatments. Previous study by Lundgren and Granéli^20^ indicated that reduced defense of *P. globosa* occurred under nutrient sufficient and P-deficient conditions. The expression of defensive trait under low nutrient condition suggested that grazing risk was a stronger selection pressure than resource availability.

Increased partitioning of cells to colonial form has been considered as another induced defense response in *P. globosa* against protozoan grazers^6^,^19^. When *P. globosa* was exposed to grazing cues from *Gyrodinium dominans* and *Euplotes*, higher percentages of cells were present in colonial form^6^,^19^. This effect occurred mostly when defensive *P. globosa* grew under limiting nutrient condition in our experiments. In contrast, grazing cues associated with *Euplotes* and *O. marina* did not change the partitioning of cells between colonial and solitary forms under nutrient-sufficient condition. Our results suggested that *P. globosa* grown under nutrient-deficient conditions had greater defensive responses, supporting the growth–differentiation balance model, which proposes that nutrient limitation can increase allocation of resources to defense^27^. When nutrient supply is sufficient, high growth may compensate for grazing losses. But when growth is limited by nutrient availability, induced defense could help conserve biomass by relieving predation losses^28^,^29^.

Previous studies reported decreased allocation of cells to colonial form when *P. globosa* received grazing cues associated with copepods^19^,^20^. These findings are not in agreement with our results which indicated that the exposure to grazing cues from copepod increased the percentages of cells in colonial form. Tang *et al*.^7^ also showed that *P. antarctica* responded to signals released during grazing activities of natural zooplankton assemblages which were dominated by copepods by partitioning cells toward the colonial phase. Variations in grazer death and differences in experimental design could result in the discrepancies between these studies. For example, we did not find any grazer mortality during the entire experiment, whereas Tang^6^ and Lundgren and Granéli^20^ found substantial copepod death at the end of experiments. The signals associated with dead copepods probably interfere with the response of *P. globosa* to the grazing cues^19^,^20^. In the case of experimental set-up, Long *et al*.^19^ and Lundgren and Granéli^20^ used filtrate containing grazing cues to induce the defense response of *P. globosa*. However, we used diffusion incubators allowing *P. globosa* to receive continuous grazing signals^30^, which is similar to what has been employed by Tang^6^. Given that our results were similar to Tang’s observations, particularly on colony enlargement and increased percentage of cells in colony form, experimental design rather than grazer death was suggested to account for the variations in defensive response of *P. globosa*.

It is assumed that a metabolic competition for resources between growth and defense occurs because both processes are energy demanding. Allocation of limited resources to induced defense prevents the use of these resources in other fitness-relevant functions^27^. As a result, the expression of induced defense in *P. globosa* is expected to impair growth, particularly considering that the mucilaginous matrix can account for >50% of the total colonial carbon of *P. globosa*^31^,^32^,^33^. Contrary to the expectation, induced defense did not negatively influence the growth rate in *P. globosa* under nutrient-replete conditions. In fact, similar to previous findings by Tang^6^, induced defensive *P. globosa* even showed higher growth rates compared with non-induced *P. globosa*. Allocation costs of induced defense can be masked in resource rich environments^15^. The maintenance of growth and reproduction may be easily achieved and more investment in defense is possible and beneficial when conditions are favorable. Thus, high nutrients may allow *P. globosa* to activate its defense while maintaining high growth rate at the same time^14^. The increased colony formation may subsequently relieve the species from grazing pressure, which may explain why *Phaeocystis* blooms occur frequently in nutrient-enriched waters such as the South China Sea, North Sea and Ross Sea^34^,^35^,^36^(, see also review by )^2^. The failure of detecting the allocation cost suggested that induced defense may incur other costs that were not included in our studies. A potential cost is decreased nutrient uptake due to the presence of diffusive boundary layer around the colonies^37^. Furthermore, these costs are likely to be ecological relevant, including enhanced sinking rate of colonies. *P. globosa* colonies in exponential phase always exhibit negative buoyancy^38^, while giant *P. globosa* colonies collected from Vietnamese coastal waters had sinking rates as high as 516 m d^−1^^33^. The high sinking rate poses a significant challenge for the colonies to maintain their population at the sea surface^33^

We found that under low nutrient conditions, induced defense in response to copepod and heterotrophic dinoflagellate grazing cues led to decreased growth rates. The differential-allocation models predict that primary producers should experience greater costs growing under limiting resource conditions where tradeoffs with growth would have severe fitness consequences^14^,^27^. This has been shown for induced defense in terrestrial plants^39^,^40^ and freshwater algae^23^. Our study provides the first direct experimental evidence of allocation cost of induced defense in marine phytoplankton, suggesting that trade-off between induced defense and growth exists widely across ecosystems.

The response of solitary cells to the combined effects of low nutrient and grazing cues was more sensitive than colonial cells. Under low nutrient conditions, induced defensive *P. globosa* had comparable abundances of colonial cells, but lower abundances of solitary cell when compared to non-induced *P. globosa*. These results could be explained by different ecological roles played by solitary and colonial cells. Due to nutrient limitation caused by the presence of a diffusive boundary layer surrounding a colony^37^, solitary cells are considered as a better competitor than colonial cells under low nutrient conditions^41^, whereas colonial cells are better defended against grazers^9^,^42^. Grazing cues and low nutrient level had a stronger inhibitory effect on the growth of solitary cells versus of colonial cells, exhibiting the importance of defense function over growth under low nutrient condition.

We did not detect any allocation cost of induced defense in the *Euplotes* experiments, where induced defensive *P. globosa* had comparable growth rates to non-induced *P. globosa* under low nutrient conditions. This result could be attributed to the species-specific character of grazing cues, which probably resulted in different growth response of *P. globosa*. Tang^6^ found all grazing cues released from three species of grazers feeding on four species of phytoplankton resulted in significant colony enlargement in *P. globosa*, and concluded that grazing signals are non-species-specific. This non-species-specific character allows *P. globosa* to defend itself against diverse predators and adapt to various environments. We argue that grazing cues from various grazers were slightly different. Grazing cues from copepods, ciliates and dinoflagellates in our experiments led to comparable defense responses of *P. globosa* in terms of colony size, which is similar to the results of Tang’s^6^ study, but our results showed a more specific response in growth. Based on the present data we were not able to determine whether properties of grazing cues are responsible for the lack of detection of allocation costs. Our results suggested, however, that the relationship among nutrient levels and costs of induced defense may appear to be non-linear^43^. The factor may have prevented the allocation costs of induced defense from becoming apparent even under low nutrition condition^14^. Consistent with our findings, Selander *et al*.^30^ reported that growth rates of *Alexandrium minutum* cells exposed to grazing cues from copepods were not significantly reduced compared to those of non-induced *A. minutum* cells irrespective of N and P limitation.

Our studies showed that allocation costs associated with induced defense in *P. globosa* were modulated by nutrient availability. Trade-offs between growth and induced defense emerged only under low nutrient conditions. Because *Phaeocystis* blooms in nature usually follow the diatom bloom^35^,^44^, *P. globosa* is likely to encounter environments where nutrient availability is limited. Induced defense may become more costly in terms of reduced growth rates, and consequently change the relative dominance between the solitary and colonial form, as well as colony size distributions^6^. Thus, nutrient availability probably had subtle influences on trophic interactions among *Phaeocystis* and their consumers by modulating the allocation cost associated with induced defense.

## Methods

### P. globosa and grazers

A clone of *Phaeocystis globosa*, originally isolated from the South China Sea in 2009, was maintained in artificial seawater with salinity of 30 at 20° C under 100 μmol photons m^−2^ s^−1^ on a light: dark cycle of 12:12 h. The seawater was enriched with f/2 medium without silicon^45^. The stock culture was maintained in exponential growth by regular dilution with fresh f/2 medium.

The copepods *Pseudodiaptomus poplesia*, ciliate *Euplotes* sp., and heterotrophic dinoflagellate *Oxyrrhis marina* were used as grazers in the present experiments. *P. poplesia* and *Euplotes* were isolated from the South China Sea, and *O. marina* was obtained from the Shannon Point Marine Center. All grazers were grown on *Isochrysis galbana* (Prymnesiophyceae) which was also grown in f/2 medium. To empty gut contents of copepod, adult female *P. poplesia* were transferred to filtered seawater for 24 h prior to experiments. *Euplotes* and *O. marina* were not fed and placed in the dark for 72 h before the experiment. These methods assured minimal transfer of residual food into the experiments.

### Experimental procedures

The allocation costs of induced defense in *P. globosa* were assessed at two nutrient levels (Table 1): high nutrient (HN: 88 μM nitrate, 3.6 μM phosphate) and low nutrient (LN: 8.8 μM nitrate, 0.36 μM phosphate). The nitrate and phosphate concentration in HN and LN regimes fell within the range observed during *Phaeocystis* blooms in the South China Sea^46^. Prior to the experiments, solitary *P. globosa* cells were isolated by passing culture through a 10 μm nylon sieve twice under gravity^6^,^47^. The stock of *P. globosa* was acclimated to experimental nutrient conditions for 30 days prior to experiments.

Copepod-induced defense experiments were conducted in six 500-mL beakers, each containing a cage made of 50-mL centrifuge tubes (BD Biosciences, USA) covered with a 2-μm polycarbonate filter (Millipore, USA) at one end. The filter kept grazers and prey organisms inside the cages, but allowed the exchange of grazing-related chemical cues between compartments. Triplicate 300 mL of stock culture from each nutrient level with starting cell concentration of 10^3^ cells mL^−1^ were distributed into beakers, and each of the cages also received 50 mL culture. Three mature female *P. poplesia* were sorted and washed with filtered artificial seawater twice before being transferred into each of three cages. The remaining three cages containing *P. globosa* alone served as controls. All beakers were covered with plastic film, and maintained for 7 or 9 days under HN and LN conditions, respectively, at 20 °C with 100 μmol photons m^−2^ s^−1^ under a light: dark cycle of 12:12 h. To facilitate dissolved chemical cues exchange between cages and beakers, all cages were gently inverted five times daily; then all beakers were shaken gently five times by hand to keep cells suspended^30^.

To test if allocation costs were a function of grazer species, we conducted parallel experiments with protozoan grazers. *Euplotes* and *O. marina* were added to grazing cages to produce a concentration of 1 and 50 cells mL^−1^, respectively, both of which were comparable to observed field values^48^,^49^. The starting concentration of solitary *P. globosa* cells in all beakers and cages was 10^3^ cells mL^−1^ and incubations lasted for 7 or 9 days under HN and LN conditions, respectively.

### Microscopy determinations and nutrient analysis

Samples for microscopic enumeration of *O. marina* and *Euplotes*, solitary cell abundances, colony numbers, colony diameters, and cells per colony of *P. globosa* inside and outside all cages were collected after incubation with a wide-mouth pipette and preserved in 4% acid Lugol’s solution^5^. All samples were enumerated within one day of collection to prevent cellular and colony disruption. Solitary cell abundances of *P. globosa* were determined with 1 mL Sedgwick-Rafter chambers. We counted solitary cells in randomly selected field of view using Nikon inverted microscope (200× magnification) until 600 cells were counted per replicate. Protozoan grazer numbers, colony concentration, colony size, and cells per colony in *P. globosa* were measured in 24-well plates using Nikon inverted microscope with a calibrated micro-ruler^6^,^50^,^51^. Twenty to 30 colonies were randomly chosen from each sample to determine colony diameter and colony cell abundance^5^. Growth rates (*μ*, d^−1^) were calculated by the equation:


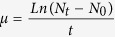


Where *t* (d) is the duration of the experiment, *N*_*t*_ and *N*_*0*_ are final and initial total cell abundance (solitary + colonial, cells mL^−1^). The reduction in growth rate in *P. globosa* exposed to grazing cues compared to controls was taken as the allocation cost of induced defense^52^,^53^. Analyses of inorganic nitrogen (nitrate and ammonium) and phosphorus inside grazing and control beakers were carried out using standard colorimetric methods^54^. The detection limits for nitrate, ammonium and phosphate were 0.02, 0.1 and 0.02 μM, respectively.

### Statistical analysis

Statistical analyses and graphical presentations were performed using Prism (v.5.0, GraphPad). All data were checked for normal distribution by means of the D’Agostino-Pearson normality test. Statistical differences in colony diameter of induced vs. non-induced *P. globosa* were assessed using a two-tailed *t*-test because the data were normally distributed. Non-parametric Mann-Whitney *U*-test was used to compare partitioning of cells, abundances of solitary and colonial cells, and growth rates of induced vs. non-induced *P. globosa* because the assumption of normality was not met even after logarithmic transformation. The significance level for all statistical tests was set a priori at a critical *P* value of 0.05.

## Additional Information

**How to cite this article**: Wang, X. *et al*. Allocation Costs Associated with Induced Defense in *Phaeocystis globosa* (Prymnesiophyceae): the Effects of Nutrient Availability. *Sci. Rep*. **5**, 10850; doi: 10.1038/srep10850 (2015).

## Figures and Tables

**Table 1:** Concentrations (μM) of dissolved nitrate, phosphate, and ammonium in *P. globosa* cultures inside grazing and control beakers at the end of grazer-induced experiments.

Nutrient concentration		*P. poplesia*		*Euplotes*		*O. marina*	
(μM)		grazing	control	grazing	control	grazing	control
HN	NO_3_^−^	56.0 ± 3.7	54.3 ± 1.8	50.3 ± 7.6	56.3 ± 7.3	61.0 ± 4.3	56.8 ± 2.6
	PO_4_^−^	1.0 ± 0.1	1.0 ± 0.1	0.6 ± 0.0	0.6 ± 0.1	1.0 ± 0.3	1.0 ± 0.2
	NH_4_^+^	0.55 ± 0.7	0.5 ± 0.3	0.35 ± 0.38	0.19 ± 0.1	0.3 ± 0.2	—
	NO_3_^−^	1.57 ± 1.9	1.17 ± 0.7	1.87 ± 0.1	1.97 ± 0.2	2.5 ± 0.3	3.2 ± 0.4
LN	PO_4_^−^	—	—	—	—	—	—
	NH_4_^+^	0.18 ± 0.06	—	0.16 ± 0.11	0.3 ± 0.2	0.13 ± 0.12	—

The detection limits for nitrate, ammonium and phosphate were 0.02, 0.1 and 0.02 μM, respectively.

-Below detection limit.

Values are mean ± 1SD. HN and LN are high and low nutrient levels, respectively.

**Table 2:** Abundances of solitary *P. globosa* cells and grazers inside grazing and control cages at the end of grazer-induced experiments.

*P. globosa* and grazers	Nutrient	*P. poplesia*		*Euplotes*sp.		*O. marina*	
		grazing	control	grazing	control	grazing	control
Abundance of solitary cells	HN	42.7 ± 21.9	160.3 ± 13.6	47.8 ± 7.9	141.0 ± 13.3	68.0 ± 17.0	185.3 ± 16.8
(×10^3^ cells mL^−1^)	LN	3.67 ± 0.9	42.0 ± 13.0	30.3 ± 5.8	100.3 ± 19.1	1.44 ± 0.9	134.3 ± 6.6
Grazers abundance	HN	0.06 ± 0.0	0	54.0 ± 8.2	0	1083.3 ± 60.5	0
(grazers mL^−1^)	LN	0.06 ± 0.0	0	20.3 ± 6.5	0	511.0 ± 101.7	0

Values are mean ± 1SD. HN and LN are high and low nutrient levels, respectively.
